# *BMC Pregnancy and Childbirth* reviewer acknowledgement 2015

**DOI:** 10.1186/s12884-016-0826-y

**Published:** 2016-02-17

**Authors:** Nawsheen Boodhun

**Affiliations:** BioMed Central, Floor 6, 236 Gray’s Inn Road, London, WC1X 8HB UK

## Abstract

The editors of *BMC Pregnancy and Childbirth* would like to thank all our reviewers who have contributed to the journal in Volume 15 (2015).

**Annika Ahman**

Sweden

**Clara Aarts**

Sweden

**Paula Abate**

Argentina

**Parvin Abedi**

Iran

**Abdulai Abubakari**

Germany

**Ganesh Acharya**

Norway

**Princess Ruhama Acheampong**

Ghana

**Princess Acheampong**

Ghana

**Mulat Adefris**

Ethiopia

**Oyelola Adegboye**

Belgium

**Oluyinka Adejumo**

USA

**Visseho Adjiwanou**

South Africa

**Annsofie Adolfsson**

Sweden

**Schadrac Agbla**

Benin

**Gabriel Agboado**

UK

**Kingsley Agho**

Australia

**Eleonora Agricola**

Italy

**Faruk Ahmed**

Australia

**Azza Ahmed**

USA

**Saifuddin Ahmed**

USA

**Ashraful (Neeloy) Alam**

Australia

**Yueping Alex Wang**

Australia

**Nagi Ali**

Sudan

**Imran Ali Khan**

Saudi Arabia

**Hanan Al-Kadri**

Saudi Arabia

**Emma Allanson**

Australia

**Rebecca Allen**

UK

**Charles Ameh**

UK

**Amanda Ampt**

Australia

**Todd Anderson**

Canada

**Ewa Andersson**

Sweden

**Prabha Andraweera**

Sri Lanka

**Aisha Andrewin**

Belize

**Rosita Aniuliene**

Lithuania

**Gifty Dufie Antwi**

Ghana

**Olatunde Aremu**

UK

**Nigel Armfield**

Australia

**Raul Artal**

USA

**Umesh Raj Aryal**

Nepal

**Nina Asplin**

Sweden

**Nega Assefa**

Ethiopia

**Roger Ayimbillah Atinga**

Ghana

**Lou Atkinson**

UK

**Carolyn Audet**

USA

**Dagfinn Aune**

UK

**Marlein Ausems**

Netherlands

**Pia Axemo**

Sweden

**Jennifer Ayton**

Australia

**Hassan Ba'Aqeel**

Saudi Arabia

**Nadia Badawi**

Australia

**Rashmi Bagga**

India

**Frank Baiden**

Ghana

**Emily Bain**

Australia

**Kathleen Baird**

Australia

**Martha Baird**

USA

**Marian Bakker**

Netherlands

**Marie-Clare Balaam**

UK

**Daynia E Ballot**

South Africa

**Julius Bamidele**

Nigeria

**Sushanta Banerjee**

India

**Aduragbemi Banke-Thomas**

UK

**Kesha Baptiste-Roberts**

USA

**Carol Barber**

New Zealand

**Flora Maria Barbosa Da Silva**

Brazil

**Kelli Barbour**

USA

**Lesley Barclay**

Australia

**Mary Barker**

UK

**Cheryl Barnabe**

Canada

**Margaret Barnes**

Australia

**Vendula Bartáková**

Czech Republic

**Subit Barua**

USA

**Hyam Bashour**

Syrian Arab Republic

**Stuart Basten**

UK

**Brian Bateman**

USA

**Sara Bayes**

Australia

**Hamideh Bayrampour**

Canada

**Alessandra Bazzano**

USA

**Arjun Bedi**

Netherlands

**Cecily Begley**

Ireland

**Loubna Belaid**

Canada

**Candice Belanoff**

USA

**Jacqueline Bell**

UK

**Katrien Benhalima**

Belgium

**Janie Benson**

USA

**Jason Bentley**

Australia

**Karen Benzies**

Canada

**Anick Berard**

Canada

**Marie Berg**

Sweden

**Howard Berger**

Canada

**Annette Bernloehr**

Germany

**Kerstin Berntorp**

Sweden

**Fabrizio Bert**

Italy

**Prem Bhandari**

USA

**Sohinee Bhattacharya**

UK

**Sanghita Bhattacharyya**

India

**Agnieszka Bianek-Bodzak**

Poland

**Jean Joel Bigna**

Cameroon

**Lorena Binfa**

Chile

**Mary Anne Biro**

Australia

**Uta Bischof**

Germany

**Concepción Blasco-Ros**

Spain

**Janusz Blaszczyk**

Poland

**Joan Bloch**

USA

**Karen Block**

Australia

**Marie Blomberg**

Sweden

**Machteld Boel**

Netherlands

**Nansi Boghossian**

USA

**Meghan Bohren**

USA

**Carolyn Bolton Moore**

Zambia

**Paula Bolton-Maggs**

UK

**Igna Bonfrer**

Netherlands

**Oleg Borisenko**

Sweden

**Renata Bortolus**

Italy

**Sabine Bousleiman**

USA

**Angela Bowen**

Canada

**Jennifer Bowen**

Australia

**Marek Brabec**

Czech Republic

**Susan Bradley**

UK

**Vivienne Brady**

Ireland

**Olivier Braissant**

Switzerland

**Stephan Brenner**

Germany

**Alexandra Brentani**

Brazil

**Paula Brentlinger**

USA

**Christian Breymann**

Switzerland

**Lynne Briggs**

Australia

**Wendy Brodribb**

Australia

**Carinne Brody**

USA

**Gabriella Broms**

Sweden

**Louise Brough**

New Zealand

**Tim Bruckner**

USA

**Ashley Bryce**

UK

**Luc Budé**

Netherlands

**Pierre Buekens**

USA

**Wilma Buffolano**

Italy

**Cynthia Bulik**

USA

**John Burgess**

Australia

**Stefanie Burghaus**

Germany

**Lisa Butler**

USA

**Alexander Butwick**

USA

**Abbey Byrne**

Australia

**Robert Cabrera**

USA

**Karin Cadwell**

USA

**Joan Cameron**

UK

**Le Ray Camille**

France

**Sandra Campbell**

Australia

**Deborah Campbell**

USA

**Simona Cardaropoli**

Italy

**Mary Carolan-Olah**

Australia

**Scala Carolina**

UK

**Andrea Cassidy-Bushrow**

USA

**Arnaud Cénac**

France

**Luisa Cescutti-Butler**

UK

**Catherine Chamberlain**

Australia

**Yiong Huak Chan**

Singapore

**Edwin Chandraharan**

UK

**Yao-Lung Chang**

Taiwan

**Jen Jen Chang**

USA

**Anne Chantry**

France

**Katie Chaput**

Canada

**Madleen Chassang**

France

**Sarika Chaturvedi**

India

**Sanjukta Chaudhuri**

USA

**Shalini Chawla**

UK

**Dong-Bao Chen**

USA

**Brian Chen**

USA

**Kate Cheney**

Australia

**Andreas Chiabi**

Cameroon

**Matthew Chico**

UK

**Jobiba Chinkhumba**

Malawi

**Joanne Chiwaula**

Ghana

**Bishnu Choulagai**

Nepal

**Mayora Chrispus**

Uganda

**Jones Chrissie**

UK

**Tereza Cindrova-Davies**

UK

**Ernestina Coast**

UK

**Judy Slome Cohain**

Israel

**Priscilla Coleman**

USA

**Katherine Collins**

UK

**Yvette Conley**

USA

**Cynthia D Connelly**

USA

**Amanda Cooklin**

Australia

**Robert Coombs**

UK

**Diane Cooper**

South Africa

**Enoka Corea**

Sri Lanka

**Denise Cote-Arsenault**

USA

**Kim Cox**

USA

**Debra Creedy**

Australia

**Ellen Cromley**

USA

**Coleen K. Cunningham**

USA

**Kenda Cunningham**

USA

**Rachel Cutting**

UK

**Allan Cyna**

Australia

**Fabricio Da Silva Costa**

Australia

**Rea Daellenbach**

New Zealand

**Darie Daemers**

Netherlands

**Rada Dagher**

USA

**Hannah Dahlen**

Australia

**Guoli Dai**

USA

**Philip Dalinjong**

Australia

**Anne Kjersti Daltveit**

Norway

**Deirdre Daly**

Ireland

**Per Damkier**

Denmark

**Kay Daniels**

USA

**Francesco D'Antonio**

Italy

**Edward T. Dassah**

Ghana

**Margie Davenport**

Canada

**Natalie Davidson**

Australia

**Miranda Davies-Tuck**

Australia

**Deborah Davis**

Australia

**Deborah Davis**

USA

**Luc De Bernis**

Ethiopia

**Caroline De Costa**

Australia

**Susie De Jersey**

Australia

**Ank De Jonge**

Netherlands

**Denhard De Smit**

Netherlands

**Sabine De Wall**

Germany

**Georgios Decavalas**

Greece

**Eugene Declercq**

USA

**Jocelyn Dejong**

Lebanon

**Anna Dencker**

Sweden

**Kebede Deribe**

Ethiopia

**Kristen Destigter**

USA

**Niveditha Devasenapathy**

India

**Nadia Diamond-Smith**

USA

**Jeffrey Dicke**

USA

**Paul Dielemans**

Malawi

**Anke Diemert**

Germany

**Lisabeth Dilalla**

USA

**Gary Dildy**

USA

**Jodie Dionne-Odom**

USA

**Jenna Dixon**

Canada

**Siobhan Dolan**

USA

**Vernon Dolinsky**

Canada

**Sara Donahue**

USA

**Amol Dongre**

India

**Jon Dorling**

UK

**Soo Downe**

UK

**Danielle Downs**

USA

**Therese Dowswell**

UK

**Ann Dozier**

USA

**Alison Drake**

USA

**Rita Driggers**

USA

**Yunden Droma**

Japan

**Rohan D'Souza**

Canada

**Guillaume Ducarme**

France

**Kirsty Dundas**

UK

**Jill Durocher**

USA

**Johannes Duvekot**

Netherlands

**Els Duysburgh**

Belgium

**Susie Dzakpasu**

Canada

**Keith Eastwood**

Australia

**Joyce Edmonds**

USA

**Andrew Edmonds**

USA

**Anbrasi Edward**

USA

**Bolaji Egbewale**

Nigeria

**Torbjørn Moe Eggebø**

Norway

**Anne Eglash**

USA

**Anne Ego**

France

**Diane Ehlers**

USA

**Clara Ejembi**

Nigeria

**Tipaya Ekalaksananan**

Thailand

**Maria Ekelin**

Sweden

**Cecilia Ekéus**

Sweden

**Anette Ekström**

Sweden

**Alison El Ayadi**

USA

**Rajavel Elango**

Canada

**Mohamed El-Gharib**

Egypt

**Kirsty Elliott-Sale**

UK

**Khalifa Elmusharaf**

Ireland

**Khalifa Elmusharaf**

Sudan

**Ruth Endacott**

UK

**Daniel Enquobahrie**

USA

**Sonja Entringer**

Germany

**Yeetey Enuameh**

Ghana

**Offer Erez**

Israel

**Carlos Escudero**

Chile

**Anne Eskild**

Norway

**Birgitta Essén**

Sweden

**Michael Evangeli**

UK

**William Evans**

USA

**E Eworuke**

USA

**Marijke Faas**

Netherlands

**Pam Factor-Litvak**

USA

**Adeniyi Fagbamigbe**

Nigeria

**Titilayo Fakeye**

Nigeria

**Abiodun Fakokunde**

UK

**Mohamed Fathalla**

Egypt

**Ahbab Mohammad Fazle Rabbi**

Bangladesh

**Denice Feig**

Canada

**Marlena Fejzo**

USA

**Anis Feki**

Switzerland

**Helen Feltovich**

USA

**Tanis Fenton**

Canada

**Shingairai Feresu**

South Africa

**Shingairai Feresu**

USA

**Nina Ferrari**

Germany

**Zach Ferraro**

Canada

**Catherine Fetherston**

Australia

**Tamara Fetters**

USA

**Andrea Fielder**

Australia

**Kenneth Finlayson**

UK

**Tabassum Firoz**

Canada

**Jane Fisher**

Australia

**Gerry Fitzgerald**

Australia

**Joel Fokom Domgue**

Cameroon

**Jane Ford**

Australia

**Della Forster**

Australia

**Edward Fottrell**

UK

**Maralyn Foureur**

Australia

**Karin Anneliese Fox**

USA

**Ana Beatriz Franco-Sena**

Brazil

**Lynn Freedman**

USA

**Atle Fretheim**

Norway

**Eleanor Friedman**

USA

**Robert Fryatt**

South Africa

**Matthew Fuller-Tyszkiewicz**

Australia

**Noya Galai**

USA

**Nicolas Galazis**

UK

**Colleen Gallagher**

USA

**Jenny Gamble**

Australia

**Hilary Gammill**

USA

**Rebecca Ganann**

Canada

**Robin Gandley**

USA

**Ashalatha Ganesh**

India

**John Kuumuori Ganle**

Ghana

**Jason Gardosi**

UK

**Michel Garenne**

France

**Ester Garne**

Denmark

**Lisa Garnweidner-Holme**

Norway

**David Garry**

USA

**Deirdre Gartland**

Australia

**Anca Gaston**

Canada

**Tania Gavidia**

Australia

**Abebaw Gebeyehu**

Ethiopia

**Alem Gebremariam**

Ethiopia

**Stacie Geller**

USA

**Compaoré Georges**

Burkina Faso

**Ioannis Germanakis**

Greece

**Melanie Gibson-Helm**

Australia

**Mark Giganti**

USA

**Christopher Gill**

USA

**Wendy Gilleard**

Australia

**Sylvie Girard**

Canada

**Carmen Giurgescu**

USA

**Norman Goco**

USA

**Sonu Goel**

India

**Laura Goetzl**

USA

**Rosa Gofin**

USA

**Robert Goldenberg**

USA

**Srinivas Goli**

India

**Bruna Gonçalves Cordeiro Da Silva**

Brazil

**Adrienne Gordon**

Australia

**Dana Gossett**

USA

**Gillian Gould**

Australia

**Aimee Grant**

UK

**Holly Grason**

USA

**Michael Gravett**

USA

**James Greenberg**

USA

**Trisha Greenhalgh**

UK

**Karleen Gribble**

Australia

**Celia Grigg**

Australia

**Rosalie Grivell**

Australia

**Chad Grotegut**

USA

**Christian Gruber**

Austria

**Imma Guerras**

Switzerland

**Elvira Guerra-Shinohara**

Brazil

**Marie-Julia Guittier**

Switzerland

**Nalika Gunawardena**

Spain

**Erica Gunderson**

USA

**Kemal Güngördük**

Turkey

**Juan De Dios Gutierrez Henares**

UK

**Rengin Guzel**

Turkey

**Wilfried Gyselaers**

Belgium

**David Haas**

USA

**Dominic Haazen**

USA

**David Hackney**

USA

**Rosalind Haddrill**

UK

**Helen Haines**

Australia

**Nafisa Halim**

USA

**Arja Halkoaho**

Finland

**Wendy Hall**

Canada

**Jennifer Hall**

USA

**Michael Hambidge**

USA

**Patti Hamilton**

USA

**Karin Hammarberg**

Australia

**Shanshan Han**

Australia

**Anette Tarp Hansen**

Denmark

**Lotte Hardman**

UK

**Donna Hartz**

Australia

**Emily Harville**

USA

**Toshiyuki Hata**

Japan

**Abigail Hatcher**

South Africa

**Yvonne Hauck**

Australia

**Claudia Hawkins**

USA

**Marquis Hawkins**

USA

**Nicola Hawley**

USA

**Mark Hayter**

UK

**Honggu He**

Singapore

**Patricia Healy**

Ireland

**Maureen Heaman**

Canada

**Alexander Heazell**

UK

**Kelsey Hegarty**

Australia

**Michael Helewa**

Canada

**Rebecca Helmreich**

USA

**Julie Hennegan**

UK

**Amanda Henry**

Australia

**Erin Henshaw**

USA

**Monika Hermann**

France

**Berenice Herve**

France

**Nicola Heslehurst**

UK

**Melissa Hill**

UK

**Zelee Hill**

UK

**Janet Hirst**

UK

**Ha Hoang**

Australia

**Stephen Hodgins**

USA

**Matthew Hoffman**

USA

**Justus Hofmeyr**

South Africa

**Theresa Hoke**

USA

**Sarah Jane Holcombe**

USA

**Jennifer Hollowell**

UK

**Sara Holton**

Australia

**Malin Holzmann**

Sweden

**Caroline Homer**

Australia

**Lucy Hope**

UK

**Kristine Hopkins**

USA

**Dell Horey**

Australia

**Bernardo Horta**

Brazil

**Jaime Horton**

USA

**Tsu-Hsin Howe**

USA

**Carl Hubel**

USA

**Tai-Ho Hung**

Taiwan

**Billie Hunter**

UK

**Darren Hutchinson**

Australia

**Marianne Hutti**

USA

**Lieven Huybregts**

Belgium

**Zubairu Iliyasu**

UK

**Por Ir**

Cambodia

**Jillian Ireland**

UK

**Farah Islam**

Canada

**Nazrul Islam**

Canada

**Ann-Charlotte Iversen**

Norway

**Jonathan Ives**

UK

**Milena Jacobs**

Australia

**B. Jager**

Netherlands

**Jennifer Jao**

USA

**Tara Jatlaoui**

USA

**Sangita Jindal**

USA

**Rosalind John**

UK

**Guro Mørk Johnsen**

Norway

**Sophia Johnson**

Australia

**Julie Jomeen**

UK

**Eleri Jones**

UK

**Catriona Jones**

UK

**K Joseph**

Canada

**Chandni Joshi**

Australia

**Andre Junqueira Caetano**

Brazil

**Alisa Kachikis**

USA

**Erkan Kalafat**

Turkey

**Yogavijayan Kandasamy**

Australia

**Lisa Kane Low**

USA

**Anil Kapur**

Denmark

**Anita Kar**

India

**Polyxeni Karakosta**

Greece

**Ali Karim**

Ethiopia

**Rajendra Karkee**

Nepal

**Bekir Karlik**

Turkey

**Spyridon Karras**

Greece

**Linda Kaste**

USA

**Jennifer Kastello**

USA

**Noriko Kato**

Japan

**Gilles Kayem**

France

**Abolanle Kayode**

Nigeria

**Xiayi Ke**

UK

**Bekana Kebede**

Ethiopia

**Bryn Kemp**

UK

**Garth Kendall**

Australia

**Holly Kennedy**

USA

**Remon Keriakos**

UK

**Sarah Finocchario Kessler**

USA

**Erum Khan**

UK

**Michelle Khan**

USA

**Sunil Khanna**

USA

**Resham Khatri**

Nepal

**Yasser Khazaal**

Switzerland

**Chong Jai Kim**

USA

**Carol Kingdon**

UK

**Dawn Kingston**

Canada

**John Kinuthia**

Kenya

**Russell Kirby**

USA

**Kristen Kjerulff**

USA

**Michael Klein**

Canada

**Marie Klingberg-Allvin**

Sweden

**Marian Knight**

UK

**Theano Kokkinaki**

Greece

**Klaas Koop**

Netherlands

**Ounjai Kor-Anantakul**

Thailand

**Irene Korstjens**

Netherlands

**Kristine Koski**

Canada

**Christopher Kovacs**

Canada

**Zoltan Kozinszky**

Sweden

**Alexander Krafft**

Switzerland

**Joan Kraft**

USA

**Ghattu V Krishnaveni**

India

**Bharati Kulkarni**

India

**Sailesh Kumar**

Australia

**Santosh Kumar**

India

**G Anil Kumar**

India

**Beenu Kushwah**

India

**Linda Kvist**

Sweden

**Barbara Kwast**

Netherlands

**Alain Labrique**

USA

**Robert Lachmann**

Germany

**Ching Tat Lai**

Australia

**Samantha Lain**

Australia

**Katariina Laine**

Norway

**Joan Lalor**

Ireland

**Yvonne Lamers**

Canada

**Ronald Lamont**

UK

**Michelle Lampl**

USA

**Stuart Lanham**

UK

**Sophia Lannon**

USA

**Hermann Lanou**

Burkina Faso

**Samia Laokri**

USA

**Malinee Laopaiboon**

Thailand

**Jacob Larkin**

USA

**Sarah Larkins**

Australia

**Elysia Larson**

USA

**Denise Lawler**

Ireland

**Beverley Lawton**

New Zealand

**Brian Layden**

USA

**Liana Leach**

Australia

**Nicky Leap**

Australia

**Charlotte Leboeuf-Yde**

Denmark

**Brigitte Leeners**

Switzerland

**Paul Leeson**

UK

**Kathryn Lemery-Chalfant**

USA

**Tiziana Leone**

UK

**Mayri Sagady Leslie**

USA

**Michael Levin**

USA

**Beth Lewis**

USA

**Zhiwen Li**

China

**Li Li**

China

**Changwei Li**

USA

**Linlin Li**

USA

**Ana Maria Linares**

USA

**Robert Liston**

Canada

**Helena Litorp**

Sweden

**Stephan Litschig**

Spain

**Tingting Liu**

USA

**Elisa Llurba**

Spain

**Yew L Lo**

Singapore

**Silvia Lobmaier**

Germany

**Robert Locke**

USA

**Ulrika Rehnström Loi**

Sweden

**Ladeisha Lombard**

Kenya

**Andres Lopez Bernal**

UK

**Jorge Lopez-Camelo**

Argentina

**Enrico Lopriore**

Netherlands

**Teresa Loureiro**

Portugal

**Julia Lowe**

Canada

**Mirjam Lukasse**

Norway

**Ingela Lundgren**

Sweden

**Riitta Luoto**

Finland

**Jacob Alexander Lykke**

Denmark

**Kimberly Ma**

USA

**Luis Machado Junior**

Brazil

**David Macintyre**

UK

**Kevin Mackway-Jones**

UK

**Bvudzai Priscilla Magadzire**

South Africa

**Everett Magann**

USA

**Per Magnus**

Norway

**Dean Maharaj**

New Zealand

**Smarajit Maiti**

India

**Eva Malacova**

Australia

**Maheen Malik**

USA

**Mats Malqvist**

Sweden

**Caroline Maltepe**

Canada

**Nataša Mandić**

Slovenia

**Gwendolin Manegold-Brauer**

Switzerland

**Bradley Manktelow**

UK

**Judith Mannien**

Netherlands

**Tanya J Marchant**

UK

**Colin Martin**

UK

**Pierre Martin-Hirsch**

UK

**Wellington Martins**

Brazil

**Katie Marvin-Dowle**

United Kingdom

**Vicki Masson**

New Zealand

**Jiji Mathews**

India

**Ilan Matok**

Israel

**Shigeki Matsubara**

Japan

**Shinya Matsuzaki**

Japan

**Anne Matthews**

Ireland

**Mandy Matthewson**

Australia

**Linda May**

USA

**Maureen Mayhew**

Canada

**Lawrence Mbuagbaw**

Cameroon

**Anthony Desmond Mccarthy**

Argentina

**Affette Mccaw-Binns**

Jamaica

**Christine Mccourt**

UK

**Judith Mccoyd**

USA

**Jean Mccrory**

USA

**Sheila Mcdonald**

Canada

**Alison Mcfadden**

UK

**Elizabeth Mcfarlane**

USA

**Rhona Mcinnes**

UK

**Meredith Mcintyre**

Australia

**Britt Mckinnon**

Canada

**Helen Mclachlan**

Australia

**Monica Mclemore**

USA

**Azar Mehrabadi**

Canada

**Yared Mekonnen**

Ethiopia

**Vn Melnikov**

Russian Federation

**Sara Meltzer**

Canada

**Ramkumar Menon**

USA

**Fiona Mensah**

UK

**Astrid Merkx**

Netherlands

**Jill Mhyre**

USA

**Ellen Mikkelsen**

Denmark

**Anne Mills**

UK

**Lesley Milne**

UK

**Abul Milton**

Australia

**Maria Amelia Miquelutti**

Brazil

**Adriana Miranda-Ribeiro**

Brazil

**Fadi Mirza**

Lebanon

**Dawn Misra**

USA

**Blandina Theophil Mmbaga**

Norway

**Markus Mohaupt**

Switzerland

**Rob Mooij**

Netherlands

**Lwendo Moonzwe Davis**

USA

**Thomas Moore**

USA

**José Morales-Roselló**

Spain

**Kate Morgaine**

UK

**Alison Morgan**

Australia

**Emmanuel Morhe**

Ghana

**Naho Morisaki**

Japan

**David Morris**

UK

**Shaine Morris**

USA

**Leonard Morssink**

Netherlands

**Andrea Mosher**

Canada

**Cheryl Moyer**

USA

**Rose Mpembeni**

Tanzania

**Mwifadhi Mrisho**

Tanzania

**Kathryn Muldoon**

Ireland

**Shama Munim**

Pakistan

**Vanessa Murphy**

Australia

**Sylvia Murphy Tighe**

Ireland

**Philippa Musoke**

Uganda

**Leslie Myatt**

USA

**Judith Myers**

Australia

**Jenny Myers**

UK

**Shamsun Nahar**

Saudi Arabia

**Manisha Nair**

UK

**Jane Nakawesi**

Uganda

**Katsuhiko Naruse**

Japan

**Mzikazi Nduna**

South Africa

**Ellen Nelissen**

Tanzania

**Britt-Ingjerd Nesheim**

Norway

**Charlotte Neumann**

USA

**James Newham**

UK

**Sam Newton**

Ghana

**Judith Nguimfack**

Senegal

**Michael Nicholl**

Australia

**Marianne Nieuwenhuijze**

Netherlands

**Anna Nikonova**

USA

**Somashekhar Nimbalkar**

India

**Tanya Nippita**

Australia

**Veeranoot Nissapatorn**

Malaysia

**Aggie Noah**

USA

**Karen Noblett**

USA

**Joy Noel-Weiss**

Canada

**Barbara Nolens**

Uganda

**Marcelo Luís Nomura**

Brazil

**Judith Noronha**

India

**Kathleen Norr**

USA

**Uchenna Nwagha**

Nigeria

**Angelo Nyamtema**

Tanzania

**Laura Oakley**

UK

**Andrew Obi**

Nigeria

**Beverley O'Brien**

Canada

**Pat O'Brien**

UK

**Louise O'Brien**

USA

**John O'Brien**

USA

**Dianne O'Connell**

Australia

**Thomas O'Connell**

USA

**Willie Oglesby**

USA

**Eric Ohuma**

UK

**Ugochukwu Vincent Okafor**

Nigeria

**Maureen Okam**

USA

**John Okanda**

Kenya

**Niran Okewole**

Nigeria

**Kehinde Okunade**

Nigeria

**Ellinor Olander**

UK

**Joann O'Leary**

USA

**Per Olofsson**

Sweden

**Beth Olson**

USA

**Bolajoko O. Olusanya**

Nigeria

**Siti Zawiah Omar**

Malaysia

**Sinead M. O'Neill**

Ireland

**Sam Ononge**

Uganda

**Dorothy Ononokpono**

Nigeria

**Uchenna Onwudiegwu**

Nigeria

**Azubuike Onyebuchi**

Nigeria

**Arwa Ooweis**

Jordan

**Baafuor Opoku**

Ghana

**Waseem Osman**

UK

**Koyejo Oyerinde**

USA

**Agnieszka Pac**

Poland

**Ana Palei**

USA

**Julie Pallant**

Australia

**Shanta Pandey**

USA

**Mary Panjari**

Australia

**Min Hae Park**

UK

**Samantha Parker**

USA

**Leslie Parker**

USA

**Crystal Patil**

USA

**Thelma Patrick**

USA

**Robert Clive Pattinson**

South Africa

**Beth Payne**

Canada

**Sallie Pearson**

Australia

**Christopher Pell**

Netherlands

**Chun Peng**

Canada

**Gavin Pereira**

USA

**Patricia Perrenoud**

Switzerland

**Eva Persson**

Sweden

**Margareta Persson**

Sweden

**Gitte Lindved Petersen**

Denmark

**Manuela Pfinder**

Germany

**Pamela Pilkington**

Australia

**M Halit Pinar**

USA

**Catherine Pirkle**

USA

**Angela Poat**

UK

**Jashvant Poeran**

USA

**Wendy Pollock**

Australia

**Albrecht Popp**

Switzerland

**Joseph Potter**

USA

**Theresa Powell**

USA

**Robert Powers**

USA

**Kathleen Powis**

USA

**Rina Pradhan**

Australia

**Ndola Prata**

USA

**Busadee Pratumvinit**

Thailand

**Walter Prendiville**

France

**Dolores Pretorius**

USA

**Holly Priddis**

Australia

**Colin Pritchard**

UK

**Victor Pulgar**

USA

**Shuby Puthussery**

UK

**Qibin Qi**

USA

**Chunfang Qiu**

USA

**Edward Quadros**

USA

**Bernardo Queiroz**

Brazil

**Ingela Radestad**

Sweden

**Sari Räisänen**

Finland

**Augustine Rajakumar**

USA

**Shanti Raman**

Australia

**Louise Randall**

Australia

**Gopi Rangan**

Australia

**Jean Rankin**

UK

**Kristen Rappazzo**

USA

**Salah Rasheed**

Egypt

**Svein Rasmussen**

Norway

**Margaret Rayman**

UK

**Juliet Rayment**

UK

**Camille Raynes-Greenow**

Australia

**Peter Rebeiro**

USA

**Maggie Redshaw**

UK

**Annette Regan**

Australia

**Donna Rennie**

Canada

**Decio Ribeiro Sarmento**

Timor-Leste

**Machteld Rijken**

Netherlands

**Marcus Rijken**

Netherlands

**Marcus Rijken**

Thailand

**Marlies Rijnders**

Netherlands

**Claire Roberts**

Australia

**Scott Roberts**

USA

**Michael Robson**

Ireland

**Lucia Rocca**

UK

**Corinne Rocca**

USA

**Lucia Rocca-Ihenacho**

UK

**Anna Joy Rogers**

USA

**Michael Ross**

USA

**Cristina Rossi**

Italy

**Heather Rowe**

Australia

**Rachel Rowe**

UK

**Sam Rowlands**

UK

**Alison Roxby**

USA

**Irene Ruengkhachorn**

Thailand

**Stephen Rulisa**

Rwanda

**Alice Rumbold**

Australia

**Rachel Rundle**

UK

**Mahama Saaka**

Ghana

**Lora Sabin**

USA

**Jayaprakash Sahoo**

India

**Yoshifumi Saisho**

Japan

**Carol Sakala**

USA

**Mihretab Melesse Salasibew**

UK

**Sarah Saleem**

Pakistan

**Jason Salemi**

USA

**Kjell Salvesen**

Norway

**Silvia Salvi**

Italy

**Juan Sanchez-Esteban**

USA

**Amarpreet Sandhu**

USA

**Valeria Sandrim**

Brazil

**Harshadkumar Sanghvi**

USA

**Ayesha Sania**

USA

**Gillian Santorelli**

UK

**Benn Sartorius**

South Africa

**Appunni Sathiya Susuman**

South Africa

**Felix Sayinzoga**

Rwanda

**Angela Scheuerle**

USA

**Crystal Schiller**

USA

**Virginia Schmied**

Australia

**Christentze Schmiegelow**

Denmark

**Francisco Schneuer**

Australia

**Ewoud Schuit**

Netherlands

**Hilary Schwandt**

USA

**Christiane Schwarz**

Germany

**Jane Scott**

Australia

**James Scott**

USA

**Nancy Scott**

USA

**Kerem Doga Seckin**

Turkey

**Olutoyin Sekoni**

Nigeria

**Daniel Sellen**

Canada

**Katherine Semrau**

USA

**Marie Seraphin**

USA

**Magdalena Serpa**

USA

**Alberto Severini**

Canada

**Haleema Shakur**

UK

**Donat Shamba**

Tanzania

**Antonia Shand**

Australia

**Sudesh Raj Sharma**

Nepal

**Sheetal Sharma**

UK

**Kathryn Sharma**

USA

**Abigail Sharpe**

UK

**Phyllis Sharps**

USA

**Jill Shawe**

UK

**Sherif Shazly**

Egypt

**Kayleigh Sheen**

UK

**Zoe Sheppard**

UK

**Jill Sherriff**

Australia

**Eiji Shibata**

Japan

**Solomon Shiferaw**

Ethiopia

**Mary Shilalukey Ngoma**

Zambia

**Brian Shine**

UK

**Shashikant Sholapurkar**

UK

**Lauren Shomaker**

USA

**Binjwala Shrestha**

Nepal

**Dimitrios Siassakos**

UK

**Estelle Monique Sidze**

Kenya

**Leigh Signal**

New Zealand

**Robert Silver**

USA

**Kari Simonsen**

USA

**Marlene Sinclair**

UK

**Deepti Singh**

India

**Kavita Singh**

USA

**Heather Sipsma**

USA

**Pauline Slade**

UK

**Nancy L Sloan**

USA

**Jennifer Slyker**

USA

**Marrit Smit**

Netherlands

**Sian Smith**

Australia

**Valerie Smith**

Ireland

**Debbie Smith**

UK

**Jeffrey Michael Smith**

USA

**Laura Sockol**

USA

**Malin Soederberg**

Sweden

**Leslie Sokolow**

USA

**Nancy Soliman**

Canada

**Andrea Solnes Miltenburg**

Tanzania

**Priya Soma-Pillay**

South Africa

**Sajid Soofi**

Pakistan

**Alexandros Sotiriadis**

UK

**David Southall**

UK

**Jonathan Spector**

USA

**Rachael Spencer**

UK

**Juliane Spiegler**

Germany

**Jiri Spilka**

Czech Republic

**Chandrashekhar Sreeramareddy**

Nepal

**Tomasina Stacey**

UK

**Anthony Staines**

Ireland

**Aleksandra Staneva**

Australia

**Joanna Stanley**

New Zealand

**Mona Stedenfeldt**

Norway

**Amie Steel**

Australia

**Phyllis Stein**

USA

**Gabrielle Stevens**

USA

**Joanna Stewart**

New Zealand

**Mary Stewart**

UK

**Elisa Stivanello**

Italy

**Kathrin Stoll**

Germany

**William Stones**

UK

**Gro Leite Størvold**

Norway

**William Story**

USA

**Patrick Stover**

USA

**Eric Strachan**

USA

**Katrine Strandberg-Laren**

Denmark

**Donna Strobino**

USA

**Christopher Sudfeld**

USA

**Colin Sullivan**

Australia

**Bo Sun**

China

**Fernanda G C Surita**

Brazil

**Fernanda Surita**

Brazil

**Rosnah Sutan**

Malaysia

**Muslim Abbas Syed**

Pakistan

**Orsolya Szenczi**

Hungary

**Angela Taft**

Australia

**Harry Tagbor**

Ghana

**Ajay Talati**

USA

**Hana Tamim**

Canada

**Peng Chiong Tan**

Malaysia

**Geok Chin Tan**

Malaysia

**Li Tang**

Australia

**Jennifer Tang**

Malawi

**Hannah Tappis**

USA

**Marie Tarrant**

Hong Kong

**Nick Tatonetti**

USA

**Paula Tavrow**

USA

**Susan Tawia**

Australia

**Lee Taylor**

Australia

**Kate Teela**

Canada

**Mathurin Tejiokem**

Cameroon

**Daniel Terry**

Australia

**Megan Teychenne**

Australia

**Zaneta Thayer**

USA

**Sudhin Thayyil**

UK

**Basky Thilaganathan**

UK

**Diana Thomas**

USA

**Susan Thompson**

UK

**Gill Thomson**

UK

**Ann Thomson**

UK

**Jim Thornton**

UK

**Andrea Tinelli**

Italy

**Christiana Titaley**

Indonesia

**William Wk To**

Hong Kong

**Angela Todd**

Australia

**Kathleen Tomsin**

Belgium

**Jocelyn Toohill**

Australia

**Maria Regina Torloni**

Brazil

**Siranda Torvaldsen**

Australia

**Sally Tracy**

Australia

**Naomi Trengove**

Australia

**Elizabeth Triche**

USA

**Helen Trottier**

Canada

**Jone Trovik**

Norway

**Lawrence Tsen**

USA

**Janet Turan**

USA

**Amy Turitz**

USA

**Deborah Turnbull**

Australia

**Marcel Twickler**

Belgium

**Zia Ul-Haq**

UK

**Priti Upadhyay**

Nepal

**Marcelo Urquia**

Canada

**Michele Usuelli**

Italy

**Bettina Utz**

Belgium

**Felipe Vadillo-Ortega**

Mexico

**Zvi Vaknin**

Israel

**Mike Van Den Hof**

Canada

**Gitsels Van Der Wal**

Netherlands

**Linde Van Genugten**

Netherlands

**Evelien Van Limbeek-Reinaerts**

Netherlands

**An-Sofie Van Parys**

Belgium

**Jos Van Roosmalen**

Netherlands

**Lenie Van Rossem**

Netherlands

**Julie Van Schalkwyk**

Canada

**Henk Van Stel**

Netherlands

**Giel Van Stralen**

Netherlands

**Edwin Van Teijlingen**

UK

**Christiaan Van Woerden**

Netherlands

**Jeroen Vanderhoeven**

USA

**Eszter Vanky**

Norway

**Peter Varga**

Hungary

**Jojy Varghese**

Australia

**Beena Varghese**

India

**Karolyn Vaughan**

Australia

**Lara Vaz**

USA

**Saraswathi Vedam**

Canada

**András Vereckei**

Hungary

**Corine Verhoeven**

Netherlands

**Georgia Verropoulou**

Greece

**Marilza Vieira Cunha Rudge**

Brazil

**Jenny Vieveen**

Netherlands

**Reeta Vijayaselvi**

India

**Dimuthu Vinayagam**

UK

**Angela Vinturache**

Canada

**Frauke Von Versen-Höynck**

Germany

**Kranti Vora**

USA

**Susan Walker**

Australia

**Lorraine Walker**

USA

**Colin Walsh**

Australia

**Denis Walsh**

UK

**Susan Walsh**

USA

**Yan-Ling Wang**

China

**Suqing Wang**

China

**Jane Warland**

Australia

**Charlotte Warren**

Kenya

**Susan Watt**

Canada

**Andrew Weeks**

UK

**Karen Weidert**

USA

**Adi Weintraub**

Israel

**Amanda Wendt**

Germany

**Benedict Weobong**

Ghana

**Mark Wetherell**

UK

**Robert White**

USA

**Clare Whitehead**

Australia

**Heather Whitford**

UK

**Melissa Whitworth**

UK

**Mariana Widmer**

Switzerland

**Therese Wiegers**

Netherlands

**Ayona Wijemanne**

UK

**Scott Wilkes**

UK

**Jeffrey Wilkinson**

USA

**Jane Willcox**

Australia

**Suzanne Willey**

Australia

**Melissa Wilson**

USA

**Ronee Wilson**

USA

**Barbara Wilson-Clay**

USA

**Christopher Winfree**

USA

**Pattanee Winichagoon**

Thailand

**Anke Witteveen**

Netherlands

**Donna Wixted**

UK

**Claire Wood**

UK

**Gang Wu**

Netherlands

**Pamela Xaverius**

USA

**Jose-Miguel Yamal**

USA

**Youli Yao**

Canada

**O. Yaw Addo**

USA

**Kojo Yeboah-Antwi**

USA

**Hung-Wen Yeh**

USA

**Jane Yelland**

Australia

**Jeanine Young**

Australia

**Sera Young**

USA

**Juping Yu**

UK

**Antonella Zambon**

Italy

**Gerald Zavorsky**

USA

**Phyllis Zelkowitz**

Canada

**Yujia Zhang**

United States

**Qiu-Yue Zhong**

USA

**Patti Zuzelo**

USA

